# Signal separation of simultaneous dual-tracer PET imaging based on global spatial information and channel attention

**DOI:** 10.1186/s40658-024-00649-9

**Published:** 2024-05-29

**Authors:** Jingwan Fang, Fuzhen Zeng, Huafeng Liu

**Affiliations:** State Key Laboratory of Modern Optical Instrumentation, College of Optical Science and Engineering, Zhejiang University, Hangzhou, China

**Keywords:** Dual-tracer PET, Signal separation, Reconstruction, Deep learning, Attention mechanism

## Abstract

**Background:**

Simultaneous dual-tracer positron emission tomography (PET) imaging efficiently provides more complete information for disease diagnosis. The signal separation has long been a challenge of dual-tracer PET imaging. To predict the single-tracer images, we proposed a separation network based on global spatial information and channel attention, and connected it to FBP-Net to form the FBPnet-Sep model.

**Results:**

Experiments using simulated dynamic PET data were conducted to: (1) compare the proposed FBPnet-Sep model to Sep-FBPnet model and currently existing Multi-task CNN, (2) verify the effectiveness of modules incorporated in FBPnet-Sep model, (3) investigate the generalization of FBPnet-Sep model to low-dose data, and (4) investigate the application of FBPnet-Sep model to multiple tracer combinations with decay corrections. Compared to the Sep-FBPnet model and Multi-task CNN, the FBPnet-Sep model reconstructed single-tracer images with higher structural similarity, peak signal-to-noise ratio and lower mean squared error, and reconstructed time-activity curves with lower bias and variation in most regions. Excluding the Inception or channel attention module resulted in degraded image qualities. The FBPnet-Sep model showed acceptable performance when applied to low-dose data. Additionally, it could deal with multiple tracer combinations. The qualities of predicted images, as well as the accuracy of derived time-activity curves and macro-parameters were slightly improved by incorporating a decay correction module.

**Conclusions:**

The proposed FBPnet-Sep model was considered a potential method for the reconstruction and signal separation of simultaneous dual-tracer PET imaging.

**Supplementary Information:**

The online version contains supplementary material available at 10.1186/s40658-024-00649-9.

## Introduction

Positron emission tomography (PET) is a molecular imaging technology measuring metabolic functions in vivo by radionuclide-labeled tracers. Benefiting from the development of various types of PET tracers, a growing number of biomarkers can be measured to aid in the early detection and diagnosis of diseases [1–3].

For diseases with complex pathological characteristics, measurements of more than one biomarker are needed, e.g., the amyloid-$\beta$ and tau in Alzheimer’s disease [4]. While this is currently accomplished by performing separate PET scans with different tracers [5], it is natural to think of using multiple tracers in a single scan for efficiency. However, the difficulty of signal separation in multi-tracer PET imaging has been an obstacle to its application, since all tracers emit 511-keV photons generated from the positron-electron annihilation, making it impossible to distinguish different tracers during the detection.

Signal separation, or reconstruction, of rapid dual-tracer PET imaging has been studied extensively. Except a small portion of studies using special tracers that emit additional prompt $\gamma$-rays [6–9], the main idea of most separation approaches is to make use of distinct temporal characteristics of different tracers to separate the mixed dual-tracer signals, as reviewed in [10]. In that case, a dynamic PET scan is necessary.

Before the rising of deep learning, methods based on the difference in tracer half-lives were firstly proposed [11] and applied [12]. Although established the foundation of using temporal characteristics to separate dual-tracer signals, these methods could only be applied when tracer concentrations reached the equilibrium. Later proposed methods were mainly based on the kinetic modeling of dual-tracer time-activity curves (TACs) by parallel compartment models [13–20]. After estimating the model parameters, the single-tracer TACs could be easily recovered. Other approaches free of compartment models included principal component analysis [21], generalized factor analysis [22], basis function fitting [23], spectral analysis [24] and recurrent extreme gradient boosting [25]. Nevertheless, these methods were limited by the requirement of arterial blood sampling or staggered injection, or might be sensitive to tracer pairs and the order of injection.

The deep learning methods do not require the arterial blood sampling, and can be applied to simultaneously injected tracers. These methods can be divided into two categories. One is the indirect reconstruction methods, which firstly reconstruct the dual-tracer images by traditional algorithms, then separate the images by neural networks [26–32]. The separation can be performed to either the voxel TACs [26–30] or the dynamic image as a whole [31, 32], with the latter utilizing the spatial information in addition to the temporal information.

The other category is the direct reconstruction methods that reconstruct the single-tracer images from the dual-tracer sinogram by usually the convolutional neural networks (CNNs), like FBP-CNN [33] and Multi-task CNN [34, 35]. The reconstruction part of FBP-CNN adopted a two-dimensional convolution layer to approximate the spatial filter, and a fully-connected layer to approximate the back-projection in the traditional filtered back-projection (FBP) algorithm. And the separation part used three-dimensional (3D) convolution kernels to learn the spatiotemporal features. In the Multi-task CNN, an encoder-decoder framework was adopted instead of the reconstruction-separation framework, and the multi-task learning mechanism was incorporated. The network was fully composed of 3D convolution and deconvolution layers. However, the performance of FBP-CNN was limited by its large network scale, which was mainly caused by the fully-connected layer. Although the Multi-task CNN contained much less parameters and was therefore easier to train, it was less interpretable. Considering the limitations of FBP-CNN and Multi-task CNN, this study aimed to propose a reconstruction-separation neural network with limited number of parameters. Besides, the 3D convolution or deconvolution kernels used in both methods processed the spatiotemporal information locally. To expand the receptive field, introducing the global feature processing might be more effective than cascading the layers using small-size kernels, which was also considered in the current study.

We firstly proposed a CNN-based model for the signal separation of simultaneous dual-tracer PET imaging. In order to extract and process global features, an Inception-like module and channel attention were applied to spatial and temporal dimensions respectively. The Inception module parallelly extracts and concatenates features of different scales to expand the receptive field [36]. In its advanced version, 3$\times$3 kernels were replaced by a combination of 1$\times$3 and 3$\times$1 kernels to save the memory [37]. Motivated by these works, we included an Inception-like module to extract global spatial features by $H\times$1 and 1$\times W$ kernels, with *H* and *W* representing the height and width of the images. The attention mechanism lets neural networks automatically learn to assign different weights to features, which means learning to focus on and select important information. It can be applied to different dimensions [38–41]. Inspired by the Squeeze-and-Excitation Networks [39], we used the channel attention for the time dimension of dynamic PET data.

The proposed separation network was then cascaded with FBP-Net [42], a deep learning implementation of the FBP algorithm, generating a direct reconstruction model named FBPnet-Sep. The FBPnet-Sep model was verified by simulated dynamic PET data, and compared to Multi-task CNN [34]. Moreover, experiments were performed to verify the superiority of image separation over sinogram separation, the effectiveness of using global spatial information and channel attention, as well as the application to low-dose data and to different tracer combinations, which will be illustrated in the following sections.

## Methods

### Model of simultaneous dual-tracer PET imaging

The simultaneous dual-tracer PET imaging is modeled as follows:

1 $$\begin{aligned} S_{dual}(t) {\sim Poisson\{} G \left[ I_1(t)+I_2(t)\right] + {r_1(t) + r_2(t)\}}. \end{aligned}$$

$S_{dual}(t)$ is the dual-tracer sinogram at time *t*. $I_1(t)$ and $I_2(t)$ are the activity images of Tracer 1 and Tracer 2. *G* is the system matrix. $r_1(t)$ and $r_2(t)$ are random coincidence events. The scatter coincidence events are not considered in this study.

The values of the *j*-th pixel in a dynamic activity image *I* depend on regional physiological parameters, the activity in the blood, and the radioactive decay of the tracer:

2 $$\begin{aligned} {I^{(j)}(t)} = C_T(t; \varvec{C_P}, {\varvec{k}^{(j)}}) \cdot e^{-ln(2) t/T}. \end{aligned}$$

$C_T$ and $\varvec{C_P}$ represent undecayed radioactivity concentrations in tissue and plasma. $\varvec{k}^{(j)}$ is the physiology-related parameters in the region corresponding to the *j*-th pixel. *T* is the half-life of radioactive decay.

### Network architecture

**Fig. 1:**
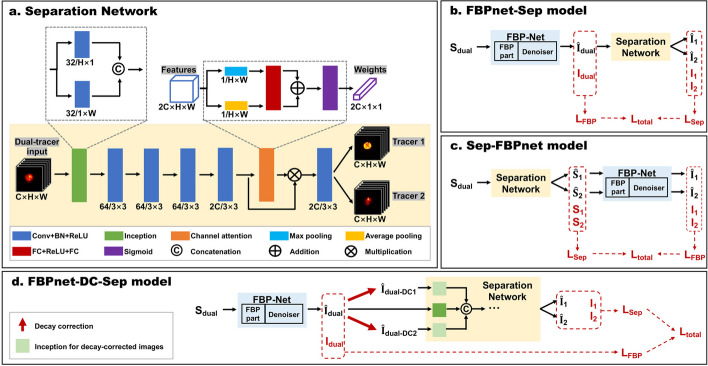
Architecture of **a** separation network, **b** FBPnet-Sep model, **c** Sep-FBPnet model and **d** FBPnet-DC-Sep model. The size and number of convolutional kernels are noted in the figure. Conv: convolutional layer, BN: batch normalization layer, ReLU: Rectified Linear Unit, FC: fully-connected layer, FBP: filtered back-projection. $S_{dual}$: dual-tracer sinogram, $S_1$: sinogram of Tracer 1, $S_2$: sinogram of Tracer 2, $I_{dual}$: dual-tracer image, $I_1$: image of Tracer 1, $I_2$: image of Tracer 2, DC: decay correction, ${\hat{I}}_{dual-DC1}$: dual-tracer image decay-corrected as Tracer 1, ${\hat{I}}_{dual-DC2}$: dual-tracer image decay-corrected as Tracer 2. ${\hat{I}}_{dual}, {\hat{I}}_1, {\hat{I}}_2, {\hat{S}}_1, {\hat{S}}_2$ are predictions of $I_{dual}, I_1, I_2, S_1, S_2$ respectively. $L_{FBP}$: loss of FBP-Net, $L_{Sep}$: loss of the separation part, $L_{total}$: total loss

In this study, we proposed a novel CNN for signal separation of simultaneous dual-tracer PET imaging, incorporating the use of global spatial information and attention mechanism. The structure of the separation network is displayed in Fig. 1a.

The input of the network is the dynamic dual-tracer image, which is in the shape of $N_b\times C\times H\times W$, where $N_b$ is the batch size, *C* is the number of channels (i.e., frames), *H* and *W* are the height and width of the image.

The network is made up of several basic convolution modules, an Inception-like module, and a channel attention module. The basic convolution module consists of a convolution layer, a batch normalization layer, and the rectified linear unit. The Inception-like module includes two basic convolution modules, which parallelly use $H\times$1 and 1$\times W$ kernels to extract features by rows and columns. The features are concatenated in channel dimension. Although different from the original Inception that used small-size kernels [37], it is still named as Inception in the current study. The channel attention module is composed of max pooling, average pooling, fully-connected layers with 2*C* neurons, and sigmoid activation function. At last, the output is separated in channel dimension to get two single-tracer dynamic images.

We further cascaded the separation network with FBP-Net to form the FBPnet-Sep model (Fig. 1b). This model firstly reconstructs and denoises the dual-tracer image by FBP-Net, then separates the image by the separation network in Fig. 1a. The FBP-Net adopts a learnable filter in the frequency domain, and maps the filtered sinogram to image by traditional back-projection without learnable parameters. Following the reconstruction, it denoises the images by a residual CNN. The details of FBP-Net can be referred to previous work of Wang and Liu [42]. For comparison, the Sep-FBPnet model (Fig. 1c) was also proposed, by which the sinogram separation was conducted before image reconstruction.

For the application to multiple tracer combinations, especially when two tracers have different half-lives, we improved the FBPnet-Sep model by adding decay corrections before the separation part, forming the FBPnet-DC-Sep model (Fig. 1d). As shown in Fig. 1d, the reconstructed dual-tracer image is decay corrected according to half-lives of two tracers respectively. The two decay-corrected images, together with the uncorrected image, are input to the separation network, and processed by different Inception modules.

### Loss functions

The loss function of a single output is a weighted summation of mean squared error (MSE) and structural similarity (SSIM) index [43] between the output and label:

3 $$\begin{aligned} L({\hat{x}},x) = \beta MSE({\hat{x}},x) + (1-\beta ) \left[ 1-SSIM({\hat{x}},x)\right] , \end{aligned}$$

where ${\hat{x}}$ is the predicted dynamic output, and *x* is the label. The coefficient $\beta$ is used to balance the MSE and SSIM.

The total loss of the entire model is composed of loss of the FBP-Net and loss of the separation part:

4 $$\begin{aligned} L_{total} = \lambda _{FBP} L_{FBP} + \lambda _{Sep} L_{Sep}. \end{aligned}$$

$\lambda _{FBP}$ and $\lambda _{Sep}$ are the weights of two losses. And the above-mentioned parameter $\beta$ is set different in reconstruction part ($\beta _{FBP}$) and separation part ($\beta _{Sep}$).

For the FBPnet-Sep (Fig. 1b) and FBPnet-DC-Sep (Fig. 1d) models, the $L_{FBP}$ serves as the auxiliary loss and $L_{Sep}$ as the main loss, which are formulated as:

5 $$\begin{aligned} L_{FBP} = L({\hat{I}}_{dual},I_{dual}), \end{aligned}$$

6 $$\begin{aligned} L_{Sep} = L({\hat{I}}_1,I_1) + L({\hat{I}}_2,I_2). \end{aligned}$$

$I_{dual}$, $I_1$ and $I_2$ are images of dual-tracer mixture, Tracer 1 and Tracer 2 respectively. Here, the coefficients in loss function are set as: $\beta _{FBP}=0.5$, $\beta _{Sep}=0.95$, $\lambda _{FBP}=1$, $\lambda _{Sep}=10$.

As for the Sep-FBPnet model (Fig. 1c), the $L_{Sep}$ serves as the auxiliary loss and $L_{FBP}$ as the main loss, which are formulated as:

7 $$\begin{aligned} L_{Sep} = L({\hat{S}}_1,S_1) + L({\hat{S}}_2,S_2), \end{aligned}$$

8 $$\begin{aligned} L_{FBP} = L({\hat{I}}_1,I_1) + L({\hat{I}}_2,I_2). \end{aligned}$$

$S_1$ and $S_2$ are sinograms of Tracer 1 and Tracer 2. Here, the coefficients are set as: $\beta _{FBP}=0.5$, $\beta _{Sep}=0.99$, $\lambda _{FBP=}1$, $\lambda _{Sep}=1$.

## Experiments

### Experimental settings

In this study, we conducted four experiments to verify the capability of the proposed method by multiple simulated datasets. The experimental settings and tracer combinations are listed in Table 1 and Table 2.

**Table 1:** Experimental settings

EXP	Dataset			Models
	Tracers	Sample size	Train:Test	
1	^18^F-FDG/^11^C-FMZ	880	800:80	FBPnet-Sep
				Sep-FBPnet
				Multi-task CNN
2	^18^F-FDG/^11^C-FMZ	880	800:80	FBPnet-Sep ablation models
3	^18^F-FDG/^11^C-FMZ, 1/2 dose	80^1^	0:240	(trained) FBPnet-Sep^2^
	^18^F-FDG/^11^C-FMZ, 1/3 dose	80^1^		
	^18^F-FDG/^11^C-FMZ, 1/5 dose	80^1^		
4	^18^F-FDG/^11^C-FMZ	880	3200:320^3^	FBPnet-Sep
	^18^F-FDG/^11^C-MET	880		FBPnet-DC-Sep
	^18^F-FDG/^18^F-AV45	880		
	^18^F-FDG/^18^F-FLT	880		

^1^Generated by down-sampling the test data of Experiment 1

^2^The FBPnet-Sep model trained in Experiment 1

^3^4800:480 for FBPnet-DC-Sep model including ^11^C-FMZ/^18^F-FDG and ^11^C-MET/^18^F-FDG

**Table 2:** Tracer combinations

Combination	Tracer 1			Tracer 2		
	Tracer	Half-life (min)	Kinetic type	Tracer	Half-life (min)	Kinetic type
1	^18^F-FDG	109.8	Irreversible	^11^C-FMZ	20.4	Reversible
2	^18^F-FDG	109.8	Irreversible	^11^C-MET	20.4	Irreversible
3	^18^F-FDG	109.8	Irreversible	^18^F-AV45	109.8	Reversible
4	^18^F-FDG	109.8	Irreversible	^18^F-FLT	109.8	Irreversible

In Experiment 1, we compared the FBPnet-Sep model which separated in the image domain with two contrast methods. One was the Sep-FBPnet model that separated in the sinogram domain, and the other was our previously proposed Multi-task CNN, which directly mapped the dual-tracer sinogram to two single-tracer images. The FBPnet-Sep model, Sep-FBPnet model and Multi-task CNN contain 0.64 M, 0.66 M and 1.31 M parameters respectively. All three models were trained and tested by simulated ^18^F-FDG/^11^C-FMZ dynamic PET data. As shown in Table 2, ^18^F-FDG and ^11^C-FMZ have different half-lives and different types of kinetic characteristics [44]. Single-tracer images inferenced by these three methods were also compared to those reconstructed from noisy single-tracer sinograms by maximum likelihood expectation maximization (MLEM) with 50 iterations.

In Experiment 2, we conducted the ablation study of FBPnet-Sep model by the same ^18^F-FDG/^11^C-FMZ dataset. The proposed FBPnet-Sep model included basic convolution modules (Conv), the Inception module (Inc) and the channel attention module (CA) in its separation part. To investigate the contribution of the Inception and channel attention modules, we compared the original FBPnet-Sep(Conv+Inc+CA) model with the FBPnet-Sep(Conv), FBPnet-Sep(Conv+Inc) and FBPnet-Sep(Conv+CA) models. In addition, to confirm the superiority of deep learning-based reconstruction over traditional iterative reconstruction, we further compared the FBPnet-Sep(Conv+Inc+CA) model with the OSEM-Sep(Conv+Inc+CA) approach, which reconstructed the dual-tracer image by ordered-subset expectation maximization (OSEM) algorithm [45] with 6 iterations and 5 subsets.

In Experiment 3, we investigated the generalization of the FBPnet-Sep model to several dose levels. The model trained in Experiment 1 was tested by ^18^F-FDG/^11^C-FMZ data of 1/2, 1/3 and 1/5 standard dose. The low-dose PET data were obtained by event reduction of test data used in Experiment 1.

In Experiment 4, data of four tracer combinations were together used to train the models, which represented different relationships between two tracers. As listed in Table 2, ^11^C-FMZ has different half-life and kinetic type from ^18^F-FDG. ^11^C-MET differs in half-life from ^18^F-FDG while ^18^F-AV45 differs in kinetic type. And ^18^F-FLT has the same half-life and kinetic type with ^18^F-FDG. To deal with multiple tracer combinations, we improved the original FBPnet-Sep model by taking the decay correction into account, getting the FBPnet-DC-Sep model. The two models were tested and compared.

### Data simulation

#### Phantoms

**Fig. 2:**
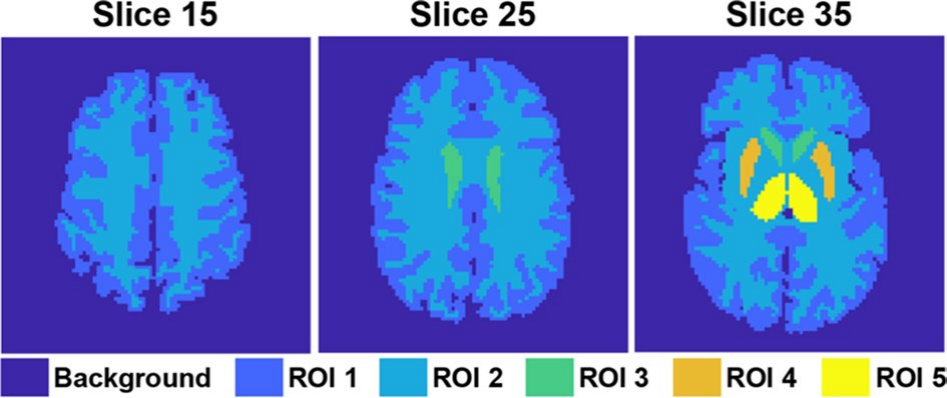
Representative brain phantoms from different slices

The two-dimensional brain phantoms used for data simulation were modified from the 3D Zubal brain phantom [46]. We chose 40 slices having different structures. The 40 phantoms are sized 128 pixels $\times$ 128 pixels, and each contains up to five regions of interest (ROIs). The average size of ROI 1 to ROI 5 are 1393, 1427, 78, 110 and 130 pixels. Representative phantoms are shown in Fig. 2.

#### Generation of dynamic activity images

The dynamic PET images were generated based on the two-tissue compartment model [44], which is a widely used kinetic model of PET tracers. It is described as:

9 $$\begin{aligned} \dfrac{dC_1(t)}{dt} =&K_1 C_P(t) - (k_2+k_3)C_1(t) + k_4 C_2(t), \end{aligned}$$

10 $$\begin{aligned} \dfrac{dC_2(t)}{dt} =&k_3 C_1(t) - k_4 C_2(t), \end{aligned}$$

11 $$\begin{aligned} C_T(t) =&C_1(t) + C_2(t). \end{aligned}$$

$C_P(t)$ is the plasma TAC, i.e., the plasma input function. $C_T(t)$ is the tissue TAC, which is the summation of TACs of two compartments, $C_1(t)$ and $C_2(t)$. The two compartments represent different metabolic states in tissue of the tracer. The rates of exchanges between different compartments (including the blood vessel) depend on the rate constants $K_1$, $k_2$, $k_3$ and $k_4$, which are also known as kinetic parameters. The values of these parameters are related to the pharmacokinetics of the tracer and physiological characteristics of its molecular target. When $k_3\gg k_4$, the transfer from Compartment 1 to Compartment 2 is regarded irreversible, the tracer is therefore considered having irreversible kinetics. Otherwise, the bound between the tracer and its target is reversible.

The Compartment Model Kinetic Analysis Tool (COMKAT) [47] provides numerical solutions of compartment models. Before solving Eqs. (9)–(11), the scanning protocol, input function and kinetic parameters of each pixel were determined. As an example, the ^18^F-FDG dynamic scan was designed with a duration of 60 min and divided into 26 frames (15 s $\times$ 8, 60 s $\times$ 8, 300 s $\times$ 10). The input function of ^18^F-FDG is modeled as:

12 $$\begin{aligned} C_P(t) = (A_1 t-A_2-A_3)e^{\lambda _1 t} + A_2 e^{\lambda _2 t} + A_3 e^{\lambda _3 t}. \end{aligned}$$

According to experimental data of human subjects [48], $A_1=851.1 \mu Ci/mL$, $A_2=21.88 \mu Ci/mL$, $A_3=20.81 \mu Ci/mL$, $\lambda _1=-4.134 min^{-1}$, $\lambda _2=-0.1191 min^{-1}$, $\lambda _3=-0.01043 min^{-1}$. In order to mimic the individual difference in physiological states, the Gaussian randomization was applied to the parameters of input function as well as the kinetic parameters of each ROI. Mean values of the parameters were referred to those reported in previous studies [18, 48], and the standard deviations were set to 10% of the mean values. Totally, 22 groups of physiological parameters were simulated. The output of compartment model was computed by COMKAT for each pixel to form the dynamic activity image of ^18^F-FDG.

Images of ^11^C-FMZ, ^11^C-MET, ^18^F-AV45 and ^18^F-FLT were generated in the same way, following the same 60-min-26-frame protocol. The input functions and the empirical values of kinetic parameters were different among tracers, which were determined according to corresponding researches [18, 48–52]. The dual-tracer activity images were obtained by adding two single-tracer images. All images were sized 128 pixels $\times$ 128 pixels $\times$ 26 frames.

#### Generation of dynamic sinograms

The dynamic single-tracer sinograms were obtained by projecting the single-tracer activity images using the Michigan Image Reconstruction Toolbox [53]. The geometry of Inveon PET/CT scanner (Siemens) was simulated. Subsequently, 20% random coincidence events were added to the projections, and the Poisson noises were simulated. The dual-tracer sinograms were the summation of two single-tracer sinograms. All sinograms were sized 128 bins $\times$ 160 angles $\times$ 26 frames.

#### Data preprocessing

For each tracer combination, a total of 880 groups (22 sets of physiological parameters $\times$ 40 phantoms) of dynamic PET data were generated. Each group of matched dynamic data consisted of the noisy dual-tracer sinogram ($S_{dual}$), two noisy single-tracer sinograms ($S_1$, $S_2$), noise-free dual-tracer activity image ($I_{dual}$) and two noise-free single-tracer activity images ($I_1$, $I_2$). In each group, sinograms were scaled by dividing max($S_{dual}$)/3, and images were scaled by dividing max($S_{dual}$)/150.

### Training details

The 880 groups of data were randomly split into training and test datasets by 10:1 according to parameter sets. In other words, all the 40 phantom slices were included in three datasets, while the physiological parameters were not repeated. The sample sizes are listed in Table 1. Note that in the training of FBPnet-DC-Sep model in Experiment 4, data was augmented by including ^11^C-FMZ/^18^F-FDG and ^11^C-MET/^18^F-FDG combinations, which were obtained by switching two tracers of ^18^F-FDG/^11^C-FMZ and ^18^F-FDG/^11^C-MET combinations.

Network training was performed on PyTorch 1.11 by a NVIDIA TITAN RTX graphics card. In the training session of each experiment, the FBP-Net and separation network were separately pretrained for 300 epochs and 100 epochs respectively, using the training dataset listed in Table 1, and both with a batch size of 4 and a learning rate of 0.0001. The entire models were trained for 100 epochs based on pre-trained network parameters and by the same dataset, with a batch size of 16 and a learning rate of 0.0001. No early stopping or regularization was used during training.

### Evaluative metrics

The performance of different methods was quantitatively evaluated by MSE, SSIM and peak signal-to-noise ratio (PSNR) between the predictions and labels. In Experiment 1, bias and standard deviation of ROI TACs and images were also evaluated.

In Experiment 4, we also estimated the macro-parameters from the predicted ROI TACs, and compared them to those derived from the label TACs. For irreversible tracers (^18^F-FDG, ^11^C-MET, ^18^F-FLT), the net uptake rate $K_i$ was estimated by Patlak plot [54]. For reversible tracers (^11^C-FMZ, ^18^F-AV45), the total distribution volume $V_T$ was estimated by Logan plot [54]. The parameters were computed in COMKAT using data of the last 10 frames. And the relative errors of the estimated macro-parameters were calculated.

## Results

### Experiment 1

**Fig. 3:**
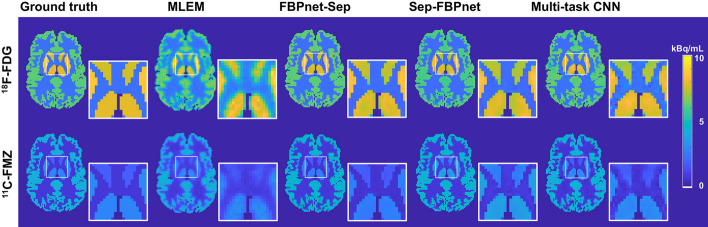
Single-tracer images predicted by different methods. All images are from Slice 30 and Frame 20

Figure 3 displays the single-tracer images reconstructed by MLEM, FBPnet-Sep model, Sep-FBPnet model and Multi-task CNN, among which the results of the proposed FBPnet-Sep model show clearest details at the boundary of ROIs.

**Fig. 4:**
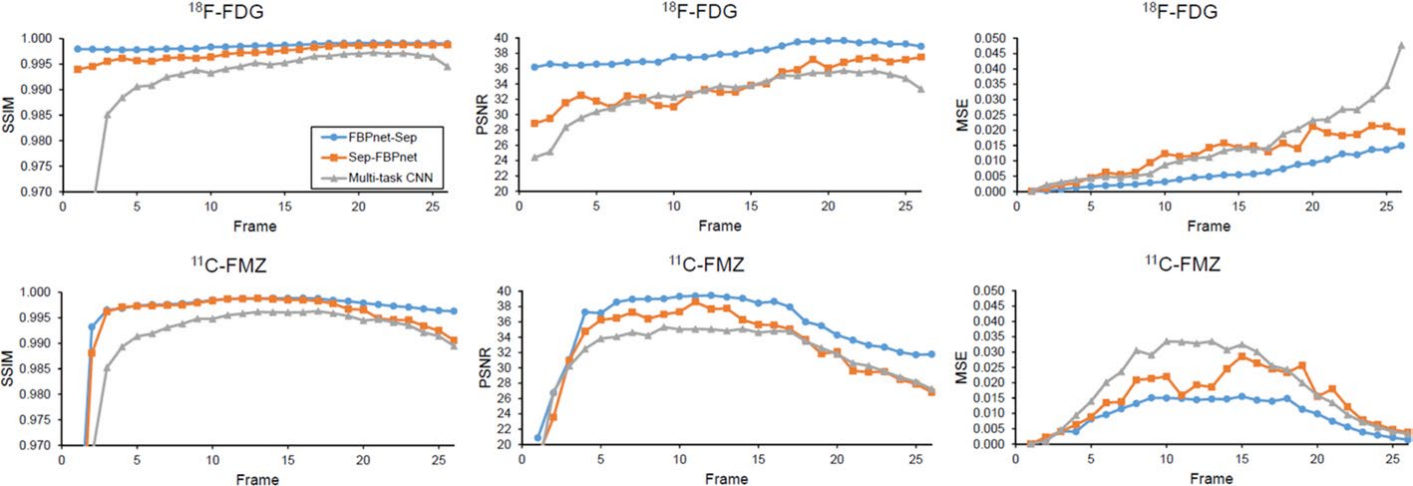
Frame-wise metrics of single-tracer images predicted by different methods. Metrics are averaged over the test dataset

Table 3 lists the mean values of the metrics. For all methods, the predictions of ^18^F-FDG images are more accurate than those of ^11^C-FMZ images. For both tracers, the FBPnet-Sep model has highest SSIM and PSNR, and lowest MSE compared to other methods. Since the noise-free single-tracer images were used as labels, all three deep-learning methods show superior performance than MLEM. In the following comparisons, MLEM was not included. Figure 4 displays the frame-wise metrics. For both ^18^F-FDG and ^11^C-FMZ, the superiority of FBPnet-Sep model lasts throughout the scan.

**Table 3:** Quantitative evaluations of the predicted images in Experiment 1

Method	^18^F-FDG			^11^C-FMZ		
	SSIM	PSNR	MSE	SSIM	PSNR	MSE
MLEM	0.9432	23.21	0.1968	0.9408	22.39	0.2388
FBPnet-Sep	**0.9985**	**37.99**	**0.0059**	**0.9958**	**35.41**	**0.0094**
Sep-FBPnet	0.9973	33.81	0.0121	0.9942	32.86	0.0149
Multi-task CNN	0.9921	32.67	0.0147	0.9891	31.91	0.0187

**Fig. 5:**
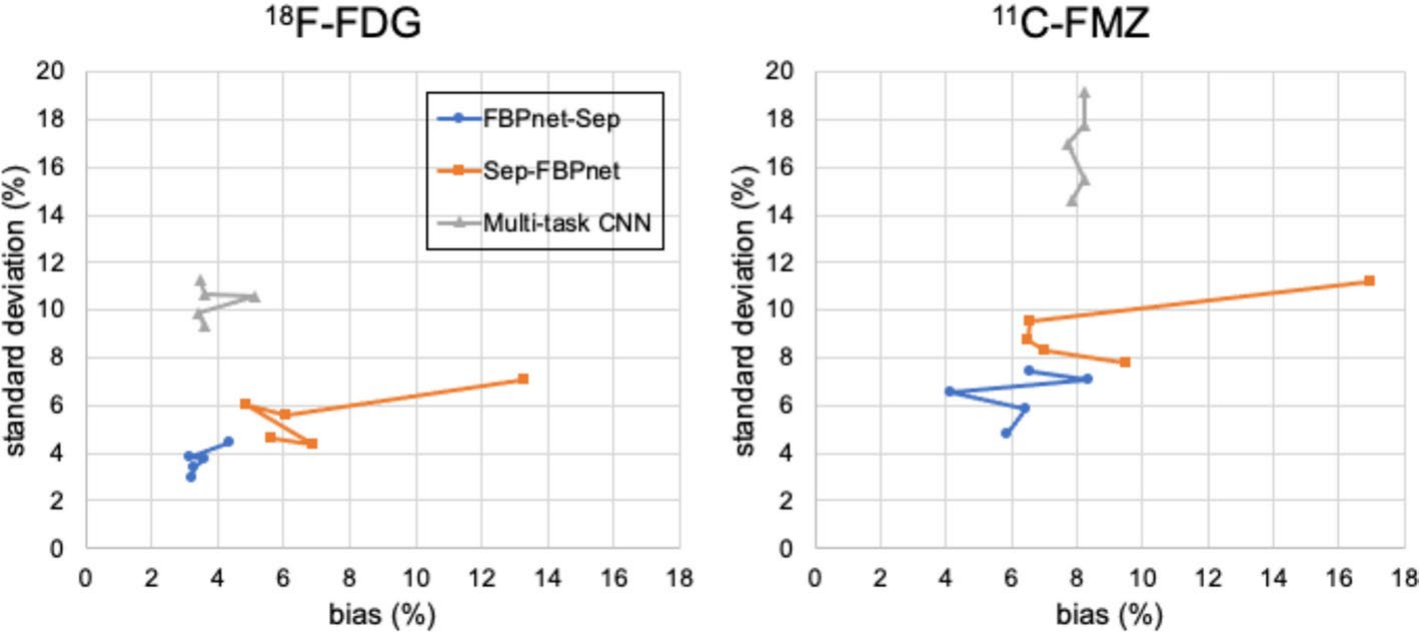
Mean bias (%) and standard deviation (%) of single-tracer images predicted by models trained after 20, 40, 60, 80 and 100 epochs

Table 4 lists the mean bias and standard deviations of ROI TACs predicted by different models. For ^18^F-FDG images, FBPnet-Sep model shows lowest bias in all ROIs except ROI 1. The other two models show high bias in small ROIs (ROI 3–5). FBPnet-Sep model reconstructs images with lowest variation in ROI 1, 2 and 4. As for ^11^C-FMZ images, the proposed FBPnet-Sep model show lowest bias in ROI 1, 2 and 3, but higher bias in ROI 4 and 5. The other two methods are highly biased in most ROIs. And the FBPnet-Sep model has lowest variation in all ROIs. Figure 5 plots the mean bias and standard deviation of images predicted by these methods, among which the FBPnet-Sep method reconstructs images with lowest bias and standard deviation.

**Table 4:** Bias (%) and standard deviation (%) of predicted ROI TACs in Experiment 1

Metric	Tracer	Model	ROI 1	ROI 2	ROI 3	ROI 4	ROI 5
Bias (%)	^18^F-FDG	FBPnet-Sep	3.0	**3.4**	**4.2**	**3.8**	**3.2**
		Sep-FBPnet	5.8	5.4	10.1	9.8	9.3
		Multi-task CNN	**2.6**	4.7	7.5	9.5	5.7
	^11^C-FMZ	FBPnet-Sep	**4.3**	**6.8**	**5.4**	10.6	9.4
		Sep-FBPnet	5.5	11.2	26.5	13.6	16.7
		Multi-task CNN	5.1	11.1	9.6	**8.7**	**9.2**
STD (%)	^18^F-FDG	FBPnet-Sep	**2.4**	**3.5**	5.7	**2.4**	3.3
		Sep-FBPnet	2.5	7.0	**2.4**	3.4	**3.0**
		Multi-task CNN	5.2	14.3	5.3	4.9	5.0
	^11^C-FMZ	FBPnet-Sep	**2.7**	**7.0**	**4.6**	**3.4**	**3.8**
		Sep-FBPnet	3.5	12.6	7.3	4.8	5.2
		Multi-task CNN	5.5	25.1	10.9	6.9	6.2

### Experiment 2

**Fig. 6:**
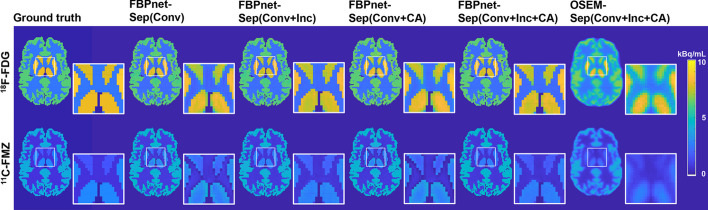
Single-tracer images predicted by different ablation models. All images are from Slice 30 and Frame 20

Figure 6 displays the representative single-tracer images predicted by FBPnet-Sep model and its ablation models. The full FBPnet-Sep(Conv+Inc+CA) model reconstructs images with best qualities, followed by FBPnet-Sep(Conv+Inc) or FBPnet-Sep(Conv+CA), while the basic FBPnet-Sep(Conv) model reconstructs images with poorer qualities. Images predicted by the OSEM-Sep(Conv+Inc+CA) approach are severely blurred.

Quantitative evaluations are listed in Table 5. For all models, the predicted ^18^F-FDG images have higher SSIM and PSNR than ^11^C-FMZ images, and have lower MSE except the FBPnet-Sep(Conv) model. For ^18^F-FDG, the FBPnet-Sep(Conv+Inc+CA) model show best performance, which is consistent with the observations in Fig. 6. For ^11^C-FMZ, the FBPnet-Sep(Conv+Inc+CA) as well as the FBPnet-Sep(Conv+Inc) models show better performance than other models.

**Table 5:** Quantitative evaluations of the predicted images in Experiment 2

Model	^18^F-FDG			^11^C-FMZ		
	SSIM	PSNR	MSE	SSIM	PSNR	MSE
FBPnet-Sep(Conv)	0.9972	34.36	0.0147	0.9929	33.58	0.0142
FBPnet-Sep(Conv+Inc)	0.9982	36.62	0.0076	0.9951	**35.81**	**0.090**
FBPnet-Sep(Conv+CA)	0.9979	36.42	0.0085	0.9956	34.93	0.0111
FBPnet-Sep(Conv+Inc+CA)	**0.9985**	**37.99**	**0.0059**	**0.9958**	35.41	0.0094
OSEM-Sep(Conv+Inc+CA)	0.9262	22.05	0.2642	0.9239	21.35	0.3267

### Experiment 3

Table 6 records the quantitative evaluations of the predicted images under different dose levels. The counts of standard dose were around 1e7. Same as the results of former experiments, the reconstructed ^18^F-FDG images are more accurate than ^11^C-FMZ images. Although slightly degrades with the decreasing dose, the qualities of reconstructed images of both tracers are acceptable even if under low-dose conditions.

**Table 6:** Quantitative evaluations of the predicted images in Experiment 3

Dose level	^18^F-FDG			^11^C-FMZ		
	SSIM	PSNR	MSE	SSIM	PSNR	MSE
Standard dose	**0.9985**	**37.99**	**0.0059**	**0.9958**	**35.41**	**0.0094**
1/2 standard dose	0.9984	37.78	0.0065	0.9953	34.94	0.0103
1/3 standard dose	0.9983	37.41	0.0076	0.9951	34.46	0.0117
1/5 standard dose	0.9980	36.84	0.0090	0.9947	33.77	0.0137

### Experiment 4

**Fig. 7:**
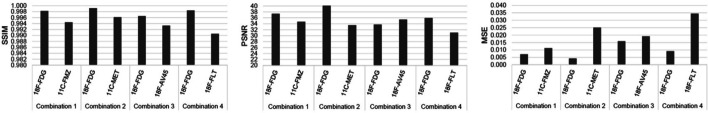
SSIM, PSNR and MSE of single-tracer images predicted by FBPnet-DC-Sep model. Metrics are averaged over the test dataset

Table 7 displays the metrics of predicted images from four tracer combinations. Both FBPnet-Sep and FBPnet-DC-Sep models were capable to deal with multiple tracer combinations. In most cases, the FBPnet-DC-Sep model exceeded the FBPnet-Sep model. Figure 7 further plots the metrics of FBPnet-DC-Sep predictions. In each tracer combination, the image qualities of ^18^F-FDG predictions were better than Tracer 2 except the PSNR of ^18^F-AV45 images. Among the other four tracers used as Tracer 2, ^18^F-FLT had lowest SSIM and PSNR, and highest MSE.

**Table 7:** Quantitative evaluations of the predicted images in Experiment 4

Tracers	Model	Tracer 1 (^18^F-FDG)			Tracer 2		
		SSIM	PSNR	MSE	SSIM	PSNR	MSE
^18^F-FDG/^11^C-FMZ	FBPnet-Sep	**0.9984**	35.69	0.0097	0.9888	30.73	0.0197
	FBPnet-DC-Sep	0.9982	**37.39**	**0.0071**	**0.9944**	**34.68**	**0.0113**
^18^F-FDG/^11^C-MET	FBPnet-Sep	0.9982	36.17	0.0093	0.9949	32.33	0.0272
	FBPnet-DC-Sep	**0.9992**	**40.57**	**0.0043**	**0.9962**	**33.54**	**0.0251**
^18^F-FDG/^18^F-AV45	FBPnet-Sep	**0.9967**	33.46	0.0166	0.9921	31.70	0.0584
	FBPnet-DC-Sep	0.9965	**33.76**	**0.0159**	**0.9933**	**35.41**	**0.0192**
^18^F-FDG/^18^F-FLT	FBPnet-Sep	0.9975	34.66	0.0113	**0.9906**	**31.56**	**0.0265**
	FBPnet-DC-Sep	**0.9984**	**35.94**	**0.0092**	0.9905	31.01	0.0345

**Fig. 8:**
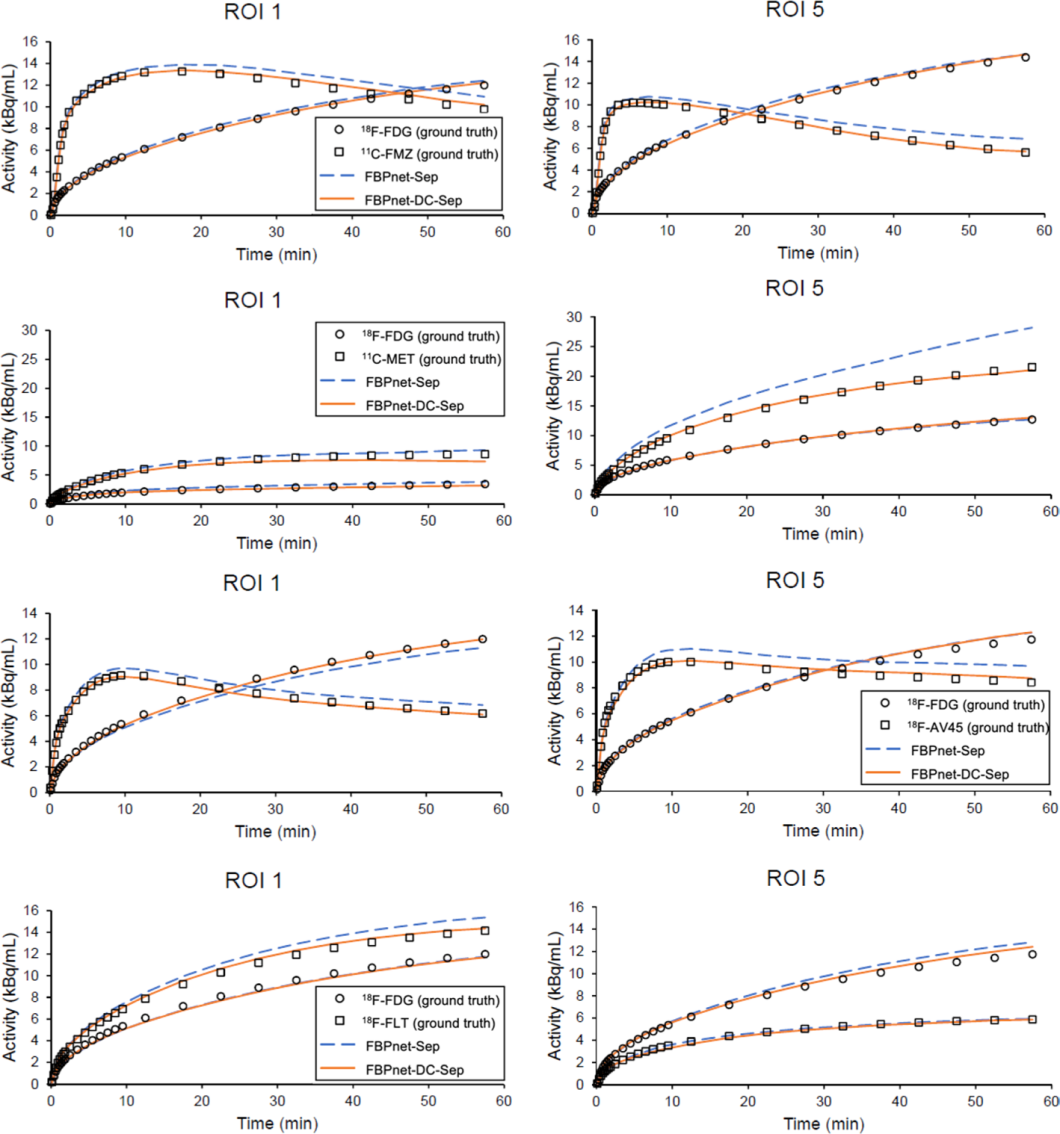
Representative ROI TACs extracted from images predicted by FBPnet-Sep and FBPnet-DC-Sep models. From top to bottom: ^18^F-FDG/^11^C-FMZ, ^18^F-FDG/^11^C-MET, ^18^F-FDG/^18^F-AV45 and ^18^F-FDG/ ^18^F-FLT combinations. From left to right: ROI 1 and ROI 5

Figure 8 plots the representative ROI TACs extracted from the predicted images. ROI 1 and ROI 5 were chosen to represent large ROIs and small ROIs respectively. In all tracer combinations, the TACs predicted by FBPnet-DC-Sep model fitted better to the ground truth than TACs predicted by FBPnet-Sep model.

**Fig. 9:**
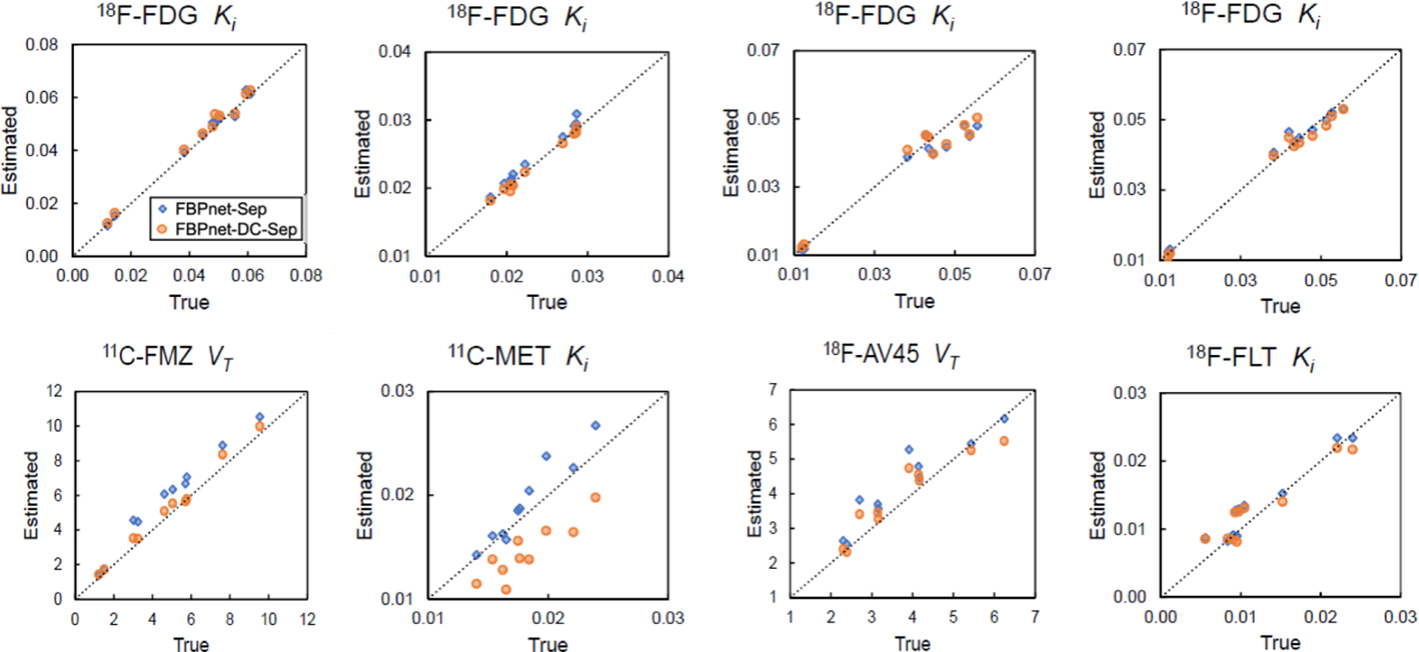
Derived macro-parameters of different tracers. From left to right: ^18^F-FDG/^11^C-FMZ, ^18^F-FDG/^11^C-MET, ^18^F-FDG/^18^F-AV45 and ^18^F-FDG/ ^18^F-FLT combinations. From top to bottom: Tracer 1 (^18^F-FDG) and Tracer 2. Parameters are averaged over the test dataset. Parameters of all ROIs are plotted in the same subplot

Macro-parameters of all tracers and all ROIs were estimated using the TACs derived from label images and predicted images. Figure 9 plots the average parameters of the test dataset. For ^18^F-FDG, the $K_i$ derived from predicted images were close to the ground truth. And no obvious difference was found between the FBPnet-DC-Sep and FBPnet-Sep models. Similar results were found in the $K_i$ of ^18^F-FLT. In the subplots of ^11^C-FMZ and ^18^F-AV45, $V_T$ derived from FBPnet-DC-Sep model were more accurate than those from FBPnet-Sep model. However, the ^11^C-MET $K_i$ derived from the FBPnet-DC-Sep model were more biased. Table 8 lists the average relative errors of the estimated macro-parameters. The results were in general consistent with Fig. 9, showing that the FBPnet-DC-Sep model was comparable to or better than FBPnet-Sep model in all tracers but ^11^C-MET. However, the accuracy of macro-parameters was sensitive to tracers and ROIs.

**Table 8:** Mean relative errors (%) of estimated macro-parameters

Tracers	Parameter	Model	ROI 1	ROI 2	ROI 3	ROI 4	ROI 5
^18^F-FDG/^11^C-FMZ	^18^F-FDG $K_i$	FBPnet-Sep	**3.3**	**2.1**	**2.9**	− 2.1	5.5
		FBPnet-DC-Sep	5.5	9.4	7.1	**0.1**	**2.6**
	^11^C-FMZ $V_T$	FBPnet-Sep	13.5	**13.9**	44.6	21.5	26.9
		FBPnet-DC-Sep	**7.1**	15.3	**12.2**	**4.4**	**5.3**
^18^F-FDG/^11^C-MET	^18^F-FDG $K_i$	FBPnet-Sep	4.6	4.7	4.4	5.4	3.4
		FBPnet-DC-Sep	**1.0**	**0.1**	**− 3.3**	**− 0.2**	**− 1.8**
	^11^C-MET $K_i$	FBPnet-Sep	**3.3**	**3.0**	**0.3**	**7.0**	**14.4**
		FBPnet-DC-Sep	− 14.6	− 16.2	− 28.0	− 22.1	− 21.7
^18^F-FDG/^18^F-AV45	^18^F-FDG $K_i$	FBPnet-Sep	**− 1.8**	**− 3.9**	− 2.7	− 15.1	− 10.8
		FBPnet-DC-Sep	4.7	4.6	**− 2.6**	**− 12.5**	**− 9.6**
	^18^F-AV45 $V_T$	FBPnet-Sep	10.7	16.8	27.2	8.9	11.5
		FBPnet-DC-Sep	**0.5**	**4.8**	**14.9**	**3.3**	**7.5**
^18^F-FDG/^18^F-FLT	^18^F-FDG $K_i$	FBPnet-Sep	3.5	**5.3**	5.7	**− 2.9**	**− 2.3**
		FBPnet-DC-Sep	**0.9**	− 5.4	**2.1**	− 3.9	− 5.6
	^18^F-FLT $K_i$	FBPnet-Sep	**1.7**	**− 2.4**	**27.7**	14.5	34.5
		FBPnet-DC-Sep	− 5.2	− 10.4	28.4	**8.6**	**31.3**

## Discussion

In this study, we proposed a CNN-based approach for the separation of simultaneously injected dual-tracer PET imaging. The network incorporated the Inception module and channel attention module. The Inception module used $H\times$1 and 1$\times W$ kernels to extract global spatial features. When applied to sinograms, it extracted features by projection angles and distance bins off the center of view. When applied to images, it extracted features by rows and columns. And the attention vector learned by channel attention could also be regarded as a global filter in time dimension. The separation network was connected to FBP-Net to predict single-tracer images from dual-tracer sinogram.

In Experiment 1, the proposed FBPnet-Sep model was firstly compared with Multi-task CNN. The Multi-task CNN had an encoder-decoder framework, while FBPnet-Sep model adopted a more interpretable reconstruction-separation framework. The results in Figs. 3, 4, 5 and Tables 3, 4 and showed that images predicted by FBPnet-Sep model had better qualities than predicted by Multi-task CNN, which might be attributed to using Inception and channel attention to process information globally rather than fully using small convolution kernels.

Additionally, we also compared image separation (FBPnet-Sep model) with sinogram separation (Sep-FBPnet model). The results showed that the FBPnet-Sep model performed better than the Sep-FBPnet model. This might be due to the differences in tasks and data distributions. In the Sep-FBPnet model, the dual-tracer sinogram $S_{dual}$ was separated to get single-tracer sinograms $S_1$ and $S_2$, both containing noises. It was beyond the capability of the model to separate the random noises. Apart from that, $S_1$ and $S_2$ were from different distributions. When they were simultaneously fed into the FBP-Net, the distribution of input became decentralized, making reconstruction more difficult. Contrarily in the FBPnet-Sep model, the FBP-Net reconstructed dual-tracer image $I_{dual}$ from $S_{dual}$, of which the distribution was relatively centralized. Besides, the reconstructed $I_{dual}$ had already been denoised, which was easier for the separation network to process.

In Experiment 2, the ablation study of the FBPnet-Sep model was conducted. We first studied on the separation part. From the results in Fig. 6 and Table 5, adding Inception module or channel attention into the basic convolutional separation network improved the model performance, and incorporating both modules achieved best performance. These results demonstrated the effectiveness of the Inception and channel attention modules. In addition, we also compared the FBPnet-Sep and OSEM-Sep approaches. The obvious superiority of the former indicated the necessity of deep learning-based reconstruction instead of traditional reconstruction algorithm.

The radiation safety and restrictions on injected doses is one of the concerns in dual-tracer PET imaging. In Experiment 3, we investigated the generalization of FBPnet-Sep model to low-dose data. As shown in Table 6, the FBPnet-Sep model could directly generalize to data of 1/2, 1/3 and 1/5 standard dose without training.

According to Eq.(2), temporal changes in radioactivity concentrations depend on both the physical radioactive decay of the tracer, which is characterized by half-life, and the physiological process the tracer is involved, which is characterized by kinetic parameters. In single-tracer PET imaging, decay correction is performed during the reconstruction. In dual-tracer PET imaging, however, decay correction cannot be conducted when two tracers have different half-lives. In that case, deep learning methods for dual-tracer PET reconstruction need to learn the hidden information about radioactive decay in addition to the kinetic characteristics.

In Experiment 4, we investigated the capability of the proposed method to deal with multiple tracer combinations. To cope with tracer pairs having different half-lives, we also proposed and tested the FBPnet-DC-Sep model. As displayed in Table 7 and Fig. 7, both methods could well predict single-tracer images of multiple tracer combinations, with the FBPnet-DC-Sep model showing better performance in most cases. According to Figs. 8, 9 and Table 8, both methods could derive ROI TACs and macro-parameters with acceptable accuracy, with the FBPnet-DC-Sep performing slightly better. These results indicated the effectiveness of the decay correction module. Although the corrections were biased, the processed images provided hints of decay information, which might make it easier for the separation network to extract useful features.

The current study still had several limitations. As shown in results, even though the method could separate two tracers, the derived TACs and macro-parameters were not accurate enough, which limited the application of dual-tracer PET imaging in further quantitative analysis. In addition, due to the lack of data from real simultaneous dual-tracer PET scans, the proposed method was verified only by simulated data. The scarcity of training data from real PET scans also limits the current study as well as other deep learning-based separation methods to separate images slice by slice. Another limitation of the proposed method is that it cannot be generalized to other phantoms, as shown in the supplementary material.

The acquisition and utilizing of training data from real dual-tracer PET scans face many challenges, like the inadequacy of paired data, image misalignment and image noises. In future studies, incorporating the kinetic model into deep learning methods for dual-tracer reconstruction and separation should be taken into consideration. A hybrid of data-driven and model-driven methods can increase the network interpretability, reduce the need for training data and be less sensitive to noises. Also, it can be easily applied to the training using ROI TACs, to increase the accuracy of recovered ROI TACs and macro-parameters. Furthermore, the generalization of the reconstruction methods to data from new distributions should also be investigated.

## Conclusions

We proposed a CNN-based model incorporating global spatial information and channel attention for the signal separation of dual-tracer PET. The separation network was extended to the FBPnet-Sep model, which predicted the single-tracer images from the dual-tracer sinogram. The FBPnet-Sep model was confirmed superior to the previously proposed Multi-task CNN. The effectiveness of Inception and channel attention modules was also verified. Moreover, the FBPnet-Sep model could be applied to data of low doses or multiple tracer combinations. Therefore, the FBPnet-Sep model can be considered as a potential method for dual-tracer PET reconstruction.

### Supplementary Information

Supplementary Information.

## Data Availability

The datasets used and analysed during the current study are available from the corresponding author on reasonable request.

## Abbreviations

PET: Positron emission tomography
TAC: Time-activity curve
CNN: Convolutional neural network
FBP: Filtered back-projection
MSE: Mean squared error
SSIM: Structural similarity
MLEM: Maximum likelihood expectation maximization
OSEM: Ordered-subset expectation maximization
ROI: Region of interest
PSNR: Peak signal-to-noise ratio
