# Supplementary material for: Signal separation of simultaneous dual-tracer PET imaging based on global spatial information and channel attention
Source: EJNMMI Phys. 2024 May 29;11:47. doi: 10.1186/s40658-024-00649-9 (PMC11136940; doi:10.1186/s40658-024-00649-9)
Supplement: Supplementary file 1 — Supplementary Information. [file 40658_2024_649_MOESM1_ESM.docx]

**Signal separation of simultaneous dual-tracer PET imaging based on global spatial information and channel attention**

**Supplementary materials**

To investigate the generalization of the proposed FBPnet-Sep network to new phantoms, two testing datasets were simulated, as shown in Fig. S1. The first dataset was simulated using the rotated brain phantom, which was generated by rotating the brain phantom used in Experiment 1 to Experiment 4. The second dataset was simulated using a thorax phantom containing three ROIs. The FBPnet-Sep network and other comparative methods trained in Experiment 1 were tested by these two datasets.


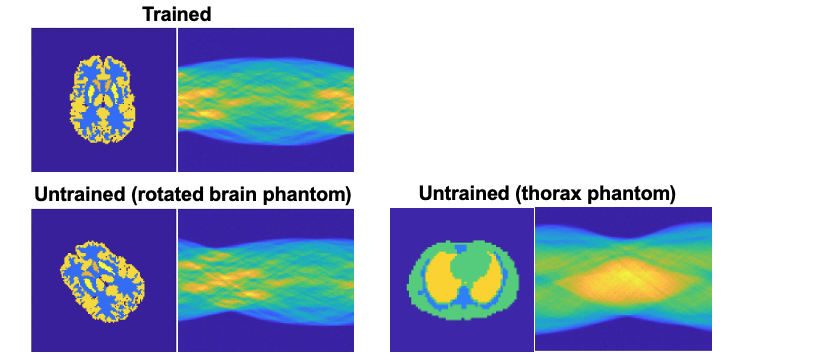


**Fig. S1.** Representative images and corresponding sinograms of training data and testing data.

Fig. S2 displays the predicted single-tracer PET images of rotated brain. FBPnet-Sep reconstructions contained severe circular artifacts that might be due to traditional back projection. By adding a mask, the structures and positions of the brain could be well reconstructed. However, the predicted tracer concentrations were inaccurate, especially in small ROIs. As for Multi-task CNN, it was interesting that the reconstructed images were translated rather than rotated, as image rotation led to sinogram translation (Fig. S1), and Multi-task CNN directly encoded the sinograms.


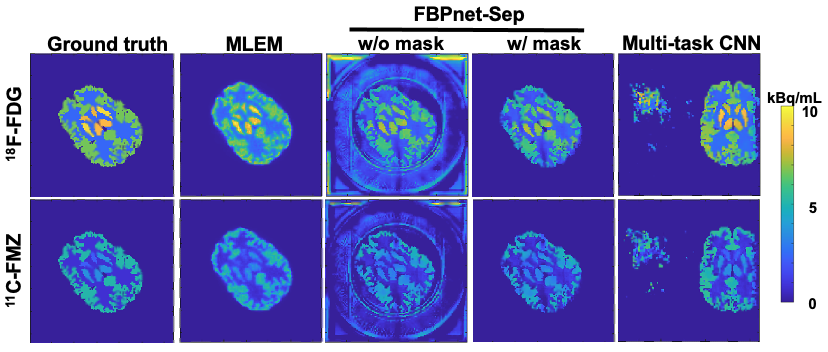


**Fig. S2.** Single-tracer images of rotated brain predicted by different methods. All images are from Slice 30 and Frame 20.

Fig. S3 shows the single-tracer PET images of thorax predicted by FBPnet-Sep method, Sep-FBPnet method and Multi-task CNN. All three methods failed to reconstruct single-tracer images. FBPnet-Sep and Sep-FBPnet methods predicted images showing some structures of the phantom, which was better than Multi-task CNN, while the circular artifacts still existed.


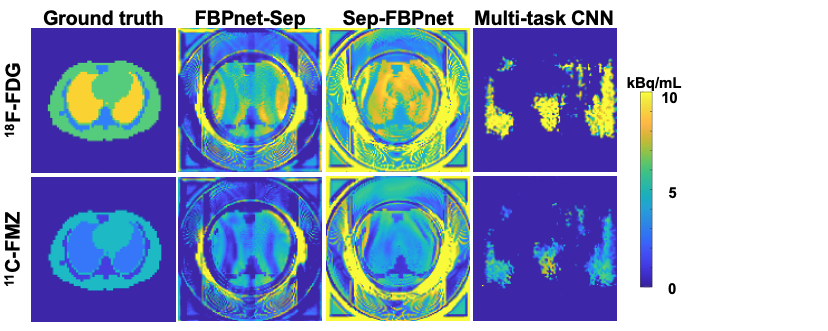


**Fig. S3.** Single-tracer images of thorax predicted by different methods. All images are from Frame 20.

According to these results, FBPnet-Sep network cannot generalize to new phantoms. Improving the reconstruction part, e.g., replacing the FBP-Net with a deep-learning implementation of iterative reconstruction algorithm, should be considered in future works.
